# The Mpumalanga Men's Study (MPMS): Results of a Baseline Biological and Behavioral HIV Surveillance Survey in Two MSM Communities in South Africa

**DOI:** 10.1371/journal.pone.0111063

**Published:** 2014-11-17

**Authors:** Tim Lane, Thomas Osmand, Alexander Marr, Starley B. Shade, Kristin Dunkle, Theodorus Sandfort, Helen Struthers, Susan Kegeles, James A. McIntyre

**Affiliations:** 1 Center for AIDS Prevention Studies, University of California San Francisco, San Francisco, California, United States of America; 2 Rollins School of Public Health, Emory University, Atlanta, Georgia, United States of America; 3 HIV Center for Clinical and Behavioral Studies, Columbia University, New York, New York, United States of America; 4 Anova Health Institute, Johannesburg, South Africa; 5 Division of Infectious Diseases & HIV Medicine, Department of Medicine, University of Cape Town, Cape Town, South Africa; 6 School of Public Health & Family Medicine, University of Cape Town, Cape Town, South Africa; University of Washington, United States of America

## Abstract

The Mpumalanga Men's Study (MPMS) is the assessment of the Project *Boithato* HIV prevention intervention for South African MSM. *Boithato* aims to increase consistent condom use, regular testing for HIV-negative MSM, and linkage to care for HIV-positive MSM. The MPMS baseline examined HIV prevalence and associated risk behaviors, and testing, care, and treatment behaviors among MSM in Gert Sibande and Ehlanzeni districts in Mpumalanga province, South Africa in order to effectively target intervention activities. We recruited 307 MSM in Gert Sibande and 298 in Ehlanzeni through respondent-driven sampling (RDS) between September 2012-March 2013. RDS-adjusted HIV prevalence estimates are 28.3% (95% CI 21.1%–35.3%) in Gert Sibande, and 13.7% (95% CI 9.1%–19.6%) in Ehlanzeni. Prevalence is significantly higher among MSM over age 25 [57.8% (95% CI 43.1%–72.9%) vs. 17.9% (95% CI 10.6%–23.9%), *P*<0.001 in Gert Sibande; 34.5% (95%CI 20.5%–56.0%) vs. 9.1% (95% CI 4.6%–13.9%), *P*<0.001 in Ehlanzeni]. In Gert Sibande, prevalence is higher among self-identified gay and transgender MSM vs. other MSM [39.3% (95%CI, 28.3%–47.9%), *P*<0.01], inconsistent condom users [38.1% (18.1%–64.2%), *P*<0.05], those with a current regular male partner [35.0% (27.1%–46.4%), P<0.05], and those with lifetime experience of intimate partner violence with men [40.4%, (95%CI 28.9%–50.9%), *P*<0.05]. Prevalence of previous HIV testing was 65.8% (95%CI 58.8%–74.0%) in Gert Sibande, and 69.3% (95%CI 61.9%–76.8%) in Ehlanzeni. Regular HIV testing was uncommon [(34.6%, (95%CI 27.9%–41.4%) in Gert Sibande; 31.0% (95%CI 24.9%–37.8%) in Ehlanzeni]. Among HIV-positive participants, few knew their status (28.1% in Gert Sibande and 14.5% in Ehlanzeni), or were appropriately linked to care (18.2% and 11.3%, respectively), or taking antiretroviral therapy (13.6% and 9.6% respectively). MPMS results demonstrate the importance of implementing interventions for MSM to increase consistent condom use, regular HIV testing, and linkage and engagement in care for HIV-infected MSM.

## Introduction

Multiple studies have confirmed the severity of the HIV epidemic among men who have sex with men (MSM) in sub-Saharan African countries. [1]–[8] In South Africa, surveys of the MSM population have found prevalence of 13.2% in Soweto, [9] 25.5% in Cape Town, [10] 27.5% in Durban, and 49.5% in greater Johannesburg. [11] HIV incidence among MSM in the region is also high. One cohort study in Kenya estimated incidence at 5.8 per 100 person-years for men who have sex with both men and women, and 35.2 per 100 person-years for men who have sex with men exclusively; [12] another found 6.8 per 100 person-years in its MSM cohort. [13] Behavioral factors may not by themselves provide a compelling explanation of high HIV prevalence and incidence of infection among MSM. [14], [15] Sexual transmission between men in the region is likely facilitated by a combination of biological, structural, and social factors, including high per-act transmission risks of anal intercourse; criminalization of homosexuality in most countries, which has been used to exclude many MSM from receiving HIV services [16]; and experiences of stigma and discrimination, particularly in the health sector, which discourage MSM from accessing available prevention and treatment services [2], [6], [11], [17]–[19].

The Republic of South Africa's explicit constitutional prohibition of discrimination on the basis of sexual orientation is unique in Africa; there are no legal barriers to providing HIV services to MSM in RSA. The World Health Organization recommends “regular” HIV testing for key affected populations, including MSM. [20] The United States Centers for Disease Control and Prevention (CDC) recommends MSM test regularly at least every six months. [21] Although there has been a dramatic expansion of treatment and prevention programming since 2009, [22] it is not known whether South African MSM currently access testing and treatment services in proportion to their need, or in sufficient numbers to check the further spread of the epidemic. In particular, critical data gaps exist for this key population on regular HIV testing, timely diagnosis of HIV infection, and timely linkage to HIV care.

To address the unmet need for targeted MSM interventions in South Africa, we established the Project Boithato HIV prevention intervention for South African MSM. Boithato is adapted from Mpowerment, a multilevel HIV prevention intervention that is effective at increasing consistent condom use among MSM. [23]–[25] As adapted, Boithato is designed to encourage greater uptake of prevention and treatment behaviors, including consistent condom use, regular HIV testing (following the CDC recommendation of every 6 months) among HIV-negative MSM, and linkage to HIV care among HIV-positive MSM.

**Figure 1:**
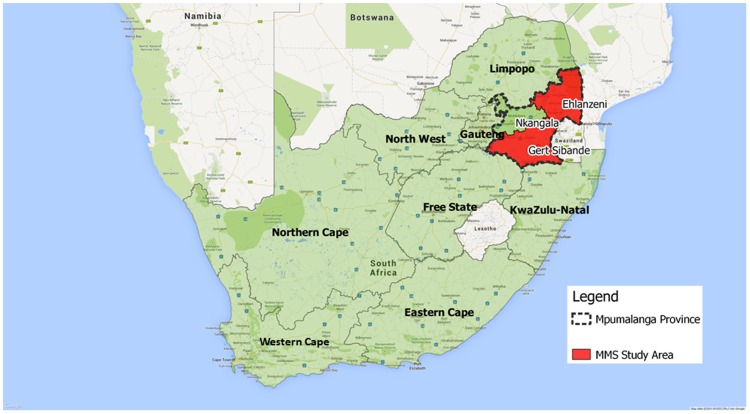
Location of Gert Sibande and Ehlanzeni District Municipalities, Mpumalanga Province, South Africa.

The Mpumalanga Men's Study is intended to evaluate the effectiveness of Boithato. MPMS is a set of serial cross-sectional, integrated biological and behavioral HIV surveillance surveys of the MSM population in the Boithato intervention community in Gert Sibande district municipality, and the comparison community in Ehlanzeni district municipality. Gert Sibande is a primarily rural district whose primary industries include agriculture, coal mining, and electricity production. Its Census 2011 population was 1.04 million. The seat of District government is Msukaligwa (Ermelo) local municipality. Ehlanzeni is also a largely rural district of 1.69 million, whose primary industries include agriculture and tourism. The seat of District government is Mbombela (Nelspruit), which is also the seat of Mpumalanga's provincial government. [26] The two districts share a border. Each is well connected to Johannesburg (roughly 200 km from Msukaligwa and 400 km from Mbombela) by well-maintained national highways. The main population centers of each district are roughly 200 km apart over relatively poorly maintained rural roads (See Figure 1 for a map of Mpumalanga).

The baseline assessment was completed between September 2012 and March 2013, prior to the Boithato intervention launch in April 2013, with follow-up assessments at 12-months and 18-months post-intervention. MSM are recruited into each wave of the assessment through respondent-driven sampling (RDS). Independent samples are recruited in each district, and each RDS recruitment wave is independent of the preceding wave. Each follow-up wave will track recapture of participants from the previous wave; staff will initiate targeted recruitment of those participants not recaptured through RDS.

In this paper, we describe the results of the MPMS baseline assessment survey of the two MSM communities. We report findings on HIV prevalence, associated risk behaviors, and health-seeking behaviors as they relate to the Boithato efficacy assessment's primary endpoints: consistent condom use, regular uptake of HIV testing, and regular uptake of care among HIV-positive MSM.

## Methods

### Recruitment and Study Procedures

Recruitment into the MPMS baseline assessment wave began simultaneously in each district and continued over 24 weeks of recruitment between September 2012 and March 2013. Following RDS recruitment procedures described by Heckathorn, [27], [28] seed participants were identified at each MPMS site based on a matrix of criteria designed to encourage the recruitment of a diverse sample of MSM from each district. These criteria included age, self-described sexual identity (e.g., gay, bisexual), educational attainment, and place of residence within the district. Each site launched recruitment with two seed participants recruited by MPMS staff, who then recruited non-seed participants, by means of study-issued coupons; these non-seed participants in turn recruited other non-seed participants via coupons, and so on. Coupons were pre-printed with an alphanumeric code consisting of a letter to identify recruitment site, a four-digit number (2000–2999) to facilitate tracking of recruitment chains, and a study phone number for recruits to call or text the local MPMS office for additional information and schedule an interview appointment. The study issued up to three recruitment coupons sequentially to each participant who completed the required study procedures, with instructions to recruit three other MSM from within their social network within the following four weeks by giving them a coupon. Monitoring of recruitment and sample characteristics at each site occurred on a weekly basis. Staff purposefully recruited two seeds to launch recruitment at each site, and additional seeds as needed based on weekly assessments of sample diversity with respect to the demographic variables of interest listed above. In total, 8 seeds were recruited in Gert Sibande, and 11 in Ehlanzeni. Participants received their choice of a mobile phone air-time voucher or a supermarket gift voucher valued at ZAR30 (US$3.00) for each participant successfully recruited and enrolled.

MSM over the age of 18 in possession of a valid study coupon, who lived, worked, or socialized with other MSM in the district where he received the coupon, and who reported anal or oral sexual activity with a man in the previous six months were eligible to enroll in the study. Recruits were screened for eligibility by MPMS staff through verification that the potential participant was in possession of a valid study coupon received from someone he knew, and by asking ten standardized screening questions about sexual activity designed to elicit answers indicative of specialized knowledge of the local MSM population and its social and sexual behaviors. To prevent the possibility that ineligible individuals could be coached into providing acceptable answers, no more than four questions were asked on any given day, and the order of questions was rotated. Individuals were deemed ineligible if they were not in possession of a coupon given to them by someone they knew, if they did not report sexual activity with another man within the specified time period, or if their replies to screening questions suggested the recruit was not likely a member of the target MSM population.

Eligible recruits who wished to enroll in the study were then asked to provide their fingerprint through the use of a commercially available digital fingerprint scanner (360 Biometrics, San Jose, CA). The scanner worked with software to generate a unique, anonymous, numeric study code for each participant, which was then linked to their coupon recruitment code. This fingerprint-generated personal identification number (PID) was used both for registration and identification purposes for future study visits to collect HIV test results or secondary study incentives, and to link a participant's behavioral data to his HIV testing results.

Participants' behavioral data were collected via an interviewer-administered computer-assisted interview on an ASUS Eee PC 1011CX N2600 netbook programmed with a QDS survey (NOVA Research, Bethesda, MD). Interviews took approximately 45–60 minutes. Interviews took place primarily in English, with interviewers offering simultaneous translation into isiZulu, Siswati, or Sesotho as requested. Data was collected over ten domains including: demographic characteristics and RDS-specific network size questions; condom use self-efficacy and safer sex social norms; perceived community cohesion and collective power; alcohol and other drug use; sexual behaviors with up to 5 of their most recent female or male sexual partners in the prior six months; knowledge of HIV transmission and prevention; knowledge and recent history of STI symptoms; experiences of MSM- or HIV-related stigma and discrimination; intimate partner violence (IPV) with regular and non-regular sexual partners; and HIV testing and treatment history and behaviors. Participants were compensated ZAR100 (∼US$10) for their time and effort in completing the survey. Participants were then offered rapid HIV testing and the option of receiving results. Participants provided separate informed consent for HIV testing per South African law. Study staff followed South African national HIV guidelines for HIV counseling, testing, and referral services.

Return of HIV results to participants was strongly encouraged but not mandatory for purposes of survey compensation of receiving recruitment coupons. For HIV-positive participants who opted to receive their results, results disclosure, post-test counseling, and appropriate referral to care and treatment were provided immediately following the rapid tests for HIV. Anova Health Institute's “Health4Men” program (www.health4men.co.za) provided training to clinical staff at four Mpumalanga Provincial Department of Health (MPDOH) referral clinics (2 per district) on providing culturally competent HIV and STI care for MSM. All participants received information packs that included condoms, water-based lubricant packets, and contact information for local referral clinics. All participants who received HIV-positive results or who reported currently experiencing STI symptoms (e.g., urethral discharge, genital ulcer) were provided with a referral letter from the study addressed to the clinics and describing their clinical needs.

Participants received up to three study coupons to recruit members of their social network after their fingerprick for HIV rapid results, but before results disclosure for those who opted to receive their results. For those who opted not to test, recruitment coupons were issued at the conclusion of the survey.

### Measurement

The behavioral survey was based on a standardized behavioral surveillance survey for MSM which had been adapted [9], [29] and used previously with South African MSM. For this assessment, we included psychosocial measures adapted by Kegeles and colleagues for the Mpowerment assessment instrument, [23] as well as additional behavioral and community measurements appropriate to the risk behavior, HIV testing, and HIV treatment behavioral outcomes of the efficacy assessment. Measurements described below pertain only to results presented in the current analysis.

Demographic indicators: Participants reported their age, highest level of educational attainment (secondary/post-secondary education), and employment in the prior six months. Participants were asked how frequently they ran out of money for basic needs in the prior six months (never/rarely/sometimes/often/always). Participants self-identified their sexual orientation as gay, bisexual, heterosexual, transgender or woman, or other.

Behavioral and HIV indicators: Participants reported the number and types of sexual partnerships with female and male partners in the prior six months. Partner types were dichotomized as “regular” and “non-regular”. Regular partnerships with men and women were defined as “someone whom you lived with, saw a lot, or felt a special emotional connection to,” and assessed as ever had and currently has a regular partner. Transactional sex was assessed by asking whether participants had ever received from or provided to a partner any of the following in exchange for sex: money or valuable goods or services, food or shelter, or drugs or alcohol. Consistent condom use was assessed by self-report on a per-partner basis. Participants were first asked to list up to 5 partners (male or female) in the prior six months, the number of vaginal, insertive anal, or receptive anal sex acts with each partner, and the number of those acts where a condom was used (protected acts). Those reporting an equal number of sex acts and protected acts were classified as consistent condom users, those with fewer protected acts than sex acts as inconsistent condom users. Alcohol consumption was assessed by the AUDIT-C screening measure, a 3-item screen asking about alcohol use in the last year, number of alcoholic drinks consumed in a typical day of drinking, and frequency of drinking six or more drinks in one day. A score of 4 or more in males is interpreted as indicative of alcohol misuse. [30] For the current analysis, because the vast majority of participants had scores indicating misuse, we separately classified those reporting drinking six or more drinks on a typical day of drinking more than one time per month in the last year as “heavy drinkers”; those who drank less were classified as “non-heavy drinkers”. Marijuana and other substance use was assessed by asking if participants had ever used marijuana, cocaine, methamphetamine (“tik”), ecstasy, or methcathinone (“khat”) and whether they had used these in the previous six months. IPV victimization was assessed by asking whether participants had ever been physically assaulted (hit, beaten, or kicked) or sexually assaulted (coercion or rape) by male partners in the prior six months. Those who reported either experience were classified as having experienced IPV.

HIV testing history and behaviors were assessed by asking whether participants had ever tested, how many times they had tested, when they last tested, and the result of their last test. MSM who had tested more than once and who reported an HIV-negative test result at their last test, were asked to describe the frequency of HIV testing as at least once every six months, at least once annually, or less than annually. Those who reported testing at least once every six months were classified as regular testers.

**Figure 2:**
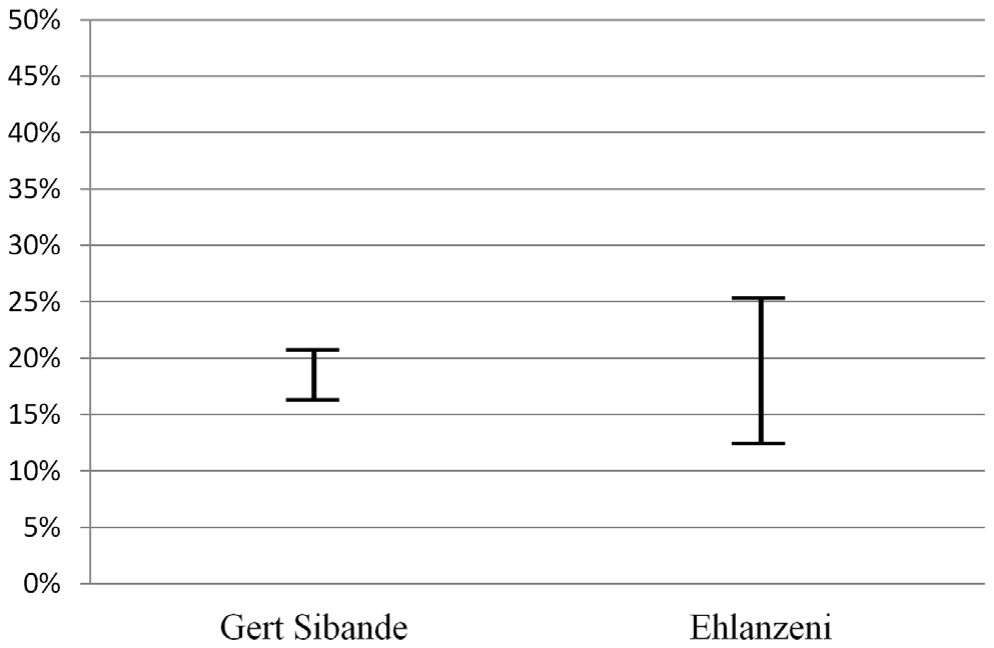
Plausibility Bounds of Undiagnosed HIV Infection. The graph indicates the upper and lower plausibility bounds of undiagnosed infection in Mpumalanga MSM. We estimate that between 16.3% and 20.6% of MSM in Gert Sibande, and 12.5% and 25.2% in Ehlanzeni, currently have an undiagnosed HIV infection. This range between lower and upper plausibility boundaries accounts for the small number of participants at each site who declined testing. The upper plausibility bound assumes all untested participants are HIV-positive, and the lower assumes all untested participants are HIV-negative.

Knowledge of HIV infection, recency of HIV infection and linkage to HIV care are composite measures of multiple survey items. Individuals whose rapid HIV test result was positive and who self-reported their HIV-positive status were classified as aware of their HIV infection; those whose rapid test result was positive and who self-reported never having tested or having received a result of HIV-negative or indeterminate on their last HIV test were classified as newly diagnosed HIV infection (also presented as undiagnosed HIV infection in Figure 2). Those individuals whose rapid test result was positive and who self-reported an HIV-negative result within the prior 12 months were classified as a recent HIV infection. Those who self-reported their last HIV test result prior to the survey as HIV-positive were classified as linked to care if they self-reported seeking medical attention for HIV within 30 days of their HIV diagnosis. Current use of antiretroviral therapy was established by self-report.

### Laboratory procedures

HIV infection was established by interpreting results of fingerprick blood samples on approved commercial rapid HIV testing kits using a parallel testing algorithm. Consenting participants were tested using EZ Trust Rapid Anti-HIV (1 &2) (CS Innovation, Singapore) [hereafter A1] and First Response HIV 1–2.0 Rapid test kits (Premier Medical Corporation, Ltd., India) [hereafter A2]. A1 and A2 non-reactive samples were interpreted as negative. A1 and A2 reactive samples were interpreted as positive. The protocol called for A1 reactive and A2 non-reactive samples to be interpreted as indeterminate and to refer participants to a local accredited laboratory for confirmatory testing following the laboratory's approved protocols for ELISA using blood collected via venipuncture.

### Statistical methods

Population prevalence estimates of demographic characteristics, HIV infection, and associated risk and health-seeking behaviors were calculated using RDSAT software version 7.1 (www.respondentdrivesampling.org). Because a small number of participants declined HIV rapid testing, undiagnosed HIV infection is presented as a range of upper and lower RDS-adjusted proportions where all non-testers were classified as HIV-positive and HIV-negative, respectively. We employed weighted logistic regression to describe the relationship between demographic and behavioral variables and HIV infection using individualized HIV outcome weights calculated in RDSAT [31], [32]. Bivariable analyses were conducted in SAS, version 9.2 (Cary, NC). Statistical associations were determined by chi-square tests at *P*<0.05 significance level. Small cell sizes prevented calculation of RDS adjusted estimates and/or 95% confidence intervals for some variables: these are noted in Tables 1–3 as “NC.” In addition, small cell sizes prevented meaningful RDS-adjusted estimates of linkage and utilization of care variables in Table 4: these results are presented as unadjusted proportions of the crude sample at each site. In our presentation of results below, we describe RDS-adjusted results in the present tense under the RDS assumption that these estimates apply to the entire population at present, and unadjusted (crude) sample results in the past tense as these refer specifically to the properties of the sample at the time it was recruited.

**Table 1:** Demographic Indicators for Mpumalanga Men's Study.

Measure		Gert Sibande (N = 307)				Ehlanzeni (N = 298)			
		Crude		Adjusted		Crude		Adjusted	
		N	%	%	95% CI	N	%	%	95% CI
Age									
18–24		199	64.8	70.4	62.8–77.5	206	69.6	72	63.1–77.4
25+		108	35.2	29.6	22.5–37.2	90	30.4	28	22.6–36.9
Highest Education Attained									
Secondary or less		199	65.5	64.2	55.1–71.4	121	41.2	47.3	38.2–54.3
Tertiary		105	34.5	35.8	28.6–45.0	173	58.8	52.7	45.7–61.8
Employment in Last 6 Months									
Had paid work		122	39.7	35.7	28.9–44.1	129	43.3	40.7	34.4–49.7
No paid work		185	60.3	64.3	55.9–71.1	169	56.7	59.3	50.3–65.6
Ran out of Money for Basic Needs in Last 6 Months									
Never/Rarely		148	48.2	45	37.6–53.1	169	56.7	45.6	38.7–54.0
Sometimes/Often/Always		159	51.8	55	46.9–62.4	129	43.3	54.4	46.0–61.3
Sexual Orientation									
Gay/Homosexual		163	53.1	46.6	38.3–55.5	151	50.7	44.5	26.6–44.1
Bisexual		128	41.7	45.4	37.3–53.7	92	30.9	35.3	36.0–51.7
Heterosexual		13	4.2	7.3	NC	7	2.4	4.5	NC
Trans/Woman		1	0.3	0.1	NC	5	1.7	0.6	NC
Other		0	0	0	NC	4	1.3	2	NC
None Reported		2	0.7	0.6	NC	39	13.1	13.1	7.7–20.3

NC  =  Not calculated due to small cell size.

**Table 2:** Behavioural and HIV Indicators in Mpumalanga Men's Study.

Measure			Gert Sibande (N = 307)				Ehlanzeni (N = 298)			
			Crude		Adjusted		Crude		Adjusted	
			N	%	%	95% CI	N	%	%	95% CI
Female Partners										
	Current regular female partner		113	38.6	40.6	32.2–48.9	67	22.7	25.7	18.05–33.2
	Past female partners		85	27.7	32.3	24.9–40.2	67	22.7	23.2	17.5–29.9
	Never had a female partner		109	35.5	27.1	19.4–35.7	161	54.6	51.1	43.4–58.2
Male Partners										
	Current regular male partner		215	70.5	66.0	58.2–73.7	224	75.7	74.2	65.9–79.9
	Past regular male partner		63	20.7	21.7	7.3–18.5	21	7.1	5.7	2.4–11.5
	Never had a male partner		27	8.9	12.2	7.3–18.5	51	17.2	20.1	14.2–27.5
Number of Female Partners in Last 6 Months										
	None		286	93.2	91.9	88.5–95.3	292	98.0	97.7	95.7–99.3
	> = 1 Partner		21	6.8	8.1	4.7–11.5	6	2.0	2.3	NC
Number of Male Partners in Last 6 Months										
	= 1 Partner		188	61.2	70.3	62.8–76.4	172	57.7	62.2	53.5–69.0
	> = 2 Partners		119	38.8	29.7	23.6–37.2	126	42.3	37.8	31.0–46.5
Transactional Sex										
	Received money, other goods		57	18.6	21.1	14.8–28.5	34	11.4	13.2	81.9–92.0
	Gave money, other goods		35	11.4	12.7	8.0–19.0	22	7.4	9.0	4.2–14.0
Condom Use with Last 5 Male Partners										
	Consistent		244	83.3	85.1	78.2–90.7	169	58.9	63.7	55.9–70.1
	Inconsistent		49	16.7	14.9	9.3–21.8	118	41.1	36.3	29.9–44.1
Drink> = 6 Drinks in One Sitting										
	Never		49	16	17.4	11.4–23.4	127	42.6	41.3	34.9–49.3
	<1/month		114	37.1	42.7	34.2–50.9	68	22.8	23.9	16.3–28.8
	2–4 times/month		141	45.9	39.9	32.5–48.5	79	26.5	26.5	20.8–33.8
	2–3 times/week		0	0	0	NC	17	5.7	6.6	3.4–10.8
	More than 4 times/week		3	1	0	NC	7	2.3	1.6	NC
Marijuana										
	Used in past 6 months		35	11.4	8.2	4.6–11.6	7	2.3	0.4	NC
Partner Violence										
	Any victimization in past 6 months		142	46.3	38.4	29.8–46.1	37	12.4	12.8	7.8–19.1
HIV Testing Behavior										
	Ever Tested in Lifetime									
		Yes	223	72.6	65.8	58.8–74.0	213	71.5	69.3	61.9–76.8
		No	84	27.4	34.2	26.0–41.2	85	28.5	30.7	23.2–38.1
	Regular Tester^1^		115	37.5	34.6	27.9–41.4	97	32.6	31.0	24.9–37.8
HIV Rapid Test Result										
	Positive		110	35.8	28.3	21.1–35.3	62	20.8	13.7	9.1–19.6
	Negative		185	60.3	67.8	61.0–75.4	197	66.1	72.4	65.2–78.5
	Not Tested		12	3.9	3.9	1.4–6.5	39	13.1	13.9	8.8–19.8

NC  =  Not calculated due to small cell size.

1 “Regular tester”:>1 lifetime HIV test & tests every 6 months.

**Table 3:** Associations between HIV infection and demographic and behavioural indicators in Mpumalanga Men's Study.

Measure			Gert Sibande (N = 307)	Ehlanzeni (N = 298)
			Adj % (95% CI)	Adj % (95% CI)
Age				
	18–24		17.9 (10.6–23.9)	9.1 (4.6–13.9)
	25+		57.8 (43.1–72.9)***	34.5 (20.5–56.0)***
Sexual Identity				
	Gay/Trans/Woman		39.3 (26.1–48.2)	13.8 (8.3–21.7)
	Bisexual		20.2 (11.4–28.8)	21.3 (10.5–31.2)
	None Reported		NC	14.4 (0.0–34.9)
Current Regular Female Partner			20.7 (8.3–28.1)	13.4 (1.0–14.6)
Male Partners				
	Current regular male partner		35.0 (27.1–46.4)**	15.0 (8.8–23.3)
	Past regular male partners		16.1 (7.2–30.5)	6.8 (0.0–21.1)
	Never had a regular male partner		7.6 (0.5–20.2)	24.5 (8.6–40.2)
Transactional Sex				
	Received money, goods, services		29.50 (15.2–49.5)	13.5 (2.9–39.6)
	Provided money, goods, services		42.70 (16.8–70.4)	NC
Condom Use with Male Partner (6 months)				
	Consistent		26.6 (19.2–35.3)	26.7 (17.2–34.3)*
	Inconsistent		38.1 (18.1–64.2)*	11.6 (6.0–19.9)
Alcohol Consumption				
	"Heavy drinkers"		30.8 (18.2–39.7)	18.2 (8.–27.5)
	Never drank heavily		22.4 (9.8–35.4)	18.7 (10.2–31.3)
Marijuana Use (6 Months)			14.4 (2.7–30.3)	NC
IPV with Male Partners			40.4 (28.9–50.9)*	NC
Negative Result on Last HIV Test			16.2 (8.8–22.9)	14.3 (3.5–17.0)

*p<0.05, **p<0.01, ***p<0.001 NC  =  Not calculated due to small cell size.

**Table 4:** Utilization of Testing and Care Among HIV+ MSM in Mpumalanga Men's Study.

Measure			Gert Sibande (N = 307)	Ehlanzeni (N = 298)
			% (n)	% (n)
HIV Rapid Test Result Positive			35.8 (110)	20.8 (62)
	Previously aware of HIV positive status		28.1 (31)	14.5 (9)
	Linked to care		18.2 (20)	11.3 (7)
	Currently taking ART		13.6 (15)	9.6 (6)
	Newly diagnosed HIV+		71.8 (79)	85.4 (53)
		Never tested for HIV	31.6 (25)	37.7 (20)
		Recent HIV infection (12 months)	22.1 (30)	39.6 (21)

NB: Figures are not RDS-adjusted estimates; all variables other than rapid results are self-reported.

### Ethics Statement

This study was approved by the University of Witwatersrand Human Research Ethic Committee (Medical) (FWA00000715) and the University of California, San Francisco Committee on Human Research (FWA00000068). All participants in this study were requested to provide written informed consent to both the behavioral questionnaire and voluntary counseling and testing (VCT). Participants who wished to remain anonymous were encouraged to use a pseudonym. All participants were able to opt out of any portion of the study.

## Results

Demographic characteristics of the two communities are presented in Table 1. The MSM population in these two Mpumalanga districts is young, largely under the age of 25. Each MSM community is characterized by relatively low levels of post-secondary education and regular employment, and nearly half of MSM in each community run short of money to meet their basic needs. The overwhelming majority of MSM describe themselves as either “gay” or “bisexual,” with similar proportions of MSM in each community self-describing their sexual identity as “gay.” Bisexual identity is more common among Gert Sibande MSM than among Ehlanzeni MSM. Few participants identify as transgender women. A sizeable proportion of MSM in Ehlanzeni do not identify as “gay,” “bisexual,” “heterosexual,” or “transgender” categories.

Key behavioral indicators are described In Table 2. Sizeable proportions of MSM have ever had a regular female partner, and most have ever had a regular male partner. Around a third of MSM had only one male partner in the prior six months, with large majorities reporting using condoms consistently with their male partners. Transactional sex is relatively common among MSM in Mpumalanga, and particularly in the Gert Sibande district where more than 1 in 5 MSM have received money, goods or services in exchange for sex. IPV is also common, particularly in Gert Sibande where nearly 2 in 5 MSM have been physically or sexually assaulted by a male partner. Substance use in the Mpumalanga MSM population is limited primarily to heavy alcohol use, where more than one-third of Ehlanzeni MSM and one-half of Gert Sibande MSM drank 6 or more alcoholic drinks in one sitting at least twice per month. Marijuana (known locally as “dagga”) is much less common. Less than one percent use stimulants including methamphetamine (“tik”), methcathinone (“khat”), cocaine, and ecstasy (data not shown).

Previous HIV testing is common among Mpumalanga MSM: around two-thirds of MSM in the two districts had tested for HIV at least once. Regular HIV testing, however, was not common, with only a third of MSM in each district reporting testing at least once every six months (See Table 2). Fifty-one MSM declined to test in the study; consequently, we estimate that at least 3.9% of MSM in Gert Sibande and 13.9% in Ehlanzeni are reluctant to test, despite a culturally competent testing environment.

We estimate HIV prevalence at 28.3% (95% CI 21.1%–35.3%) among Gert Sibande MSM, and 13.7% (95% CI 9.1–19.6%) among Ehlanzeni MSM (Table 2). HIV-positive men were disproportionately gay-identified, and 25 years of age or older—in each district, prevalence was 3 times greater in the older age group. In bivariable analysis (Table 3), HIV infection among Gert Sibande MSM was significantly associated with age 25 years or older, having a regular male partner, inconsistent condom use, and experiencing IPV from a male partner. In Ehlanzeni, age 25 years or older and consistent condom use were associated with HIV infection.

We estimate that 16.3% to 20.6% of MSM in Gert Sibande, and 12.5% to 25.2% in Ehlanzeni, currently have an undiagnosed HIV infection (Figure 2). Included in this measure were all MSM who tested positive in the survey, and those who had either never tested, or who self-reported the result of their last test as negative. (*N.B.* All participants who had self-reported the result of their last HIV test as positive provided a rapid-test sample that confirmed their HIV-positive serostatus for the study.) We present these estimates as a range between lower and upper plausibility boundaries due to the small number of participants at each site (12 and 39, respectively) who declined testing. The upper plausibility bound assumes all untested participants are HIV-positive, and the lower assumes all untested participants are HIV-negative.

We present unadjusted analysis of HIV testing utilization, linkage, and treatment among the subset of HIV-positive MSM in each sample in Table 4. No indeterminate results were observed through rapid testing. Roughly 1 in 4 HIV-positive Gert Sibande participants and 1 in 7 Ehlanzeni participants were aware of their HIV infection. Fewer than 1 in 5 HIV-positive participants were linked to care, and roughly 1 in 10 were taking antiretroviral therapy. We observed that 1 in 5 MSM in Gert Sibande, and 2 in 5 in Ehlanzeni, who had received an HIV-negative test result within the 12 months prior to the survey, were diagnosed with HIV through rapid HIV testing in the MPMS and referred to care.

## Discussion

These results demonstrate Mpumalanga MSM do not currently access HIV testing, care and treatment services in proportion to what is needed to substantially impact the course of the epidemic in this population. We observed high HIV prevalence in these communities, low uptake of regular HIV testing, and consequently a high burden of undiagnosed HIV infection. If unaddressed, this high burden of undiagnosed HIV infection will lead to increased morbidity and mortality and continued transmission of HIV within these communities of MSM. That we observed a third of the MSM in our sample who had tested negative within the last twelve moths had been infected by the time of their enrolment suggests high incidence in this population and the urgent need for expanding prevention, testing, care, and treatment programming for MSM.

HIV prevalence is markedly higher among Gert Sibande MSM than among Ehlanzeni MSM. We speculate that lower prevalence in Ehlanzeni may be attributable to the higher level of tertiary education observed in this MSM community compared to that of Gert Sibande. We further speculate that higher background HIV prevalence in Gert Sibande among the general population, (estimated at 40.5% among women from antenatal surveillance), may also contribute to higher prevalence among MSM in this district. [33] (The severity of the HIV epidemic in Gert Sibande district had not been documented at the time the sites were selected for the trial in 2010.) Nonetheless, distribution of HIV by age is markedly similar between districts: we observed prevalence roughly 3 times higher among men aged 25 and older compared to men 18–24. Additionally, in both districts HIV disproportionately affects MSM who self-identify either as gay, transsexual, or bisexual. This finding corroborates others where elevated prevalence among MSM who are gay or bisexually identified was observed. [9] It also indicates that those MSM most affected by HIV in South Africa are also those most open about their sexuality, and potentially most easily reached by existing MSM intervention models' focus on the specialized prevention needs of gay and bisexually identified MSM. Moreover, the lower observed prevalence among MSM under the age of 25 indicates that reaching younger MSM with interventions to reinforce HIV prevention behaviors including consistent condom use may yield significant results in checking the spread of HIV as MSM progress through their adult lives. Like Mpowerment, the Boithato intervention focuses explicitly on mobilizing gay- and bisexual-identified MSM to support each other in risk reduction, and has been adapted to provide a structured way of informing MSM about clinical HIV services, and for MSM to support each other to access those services they need. These would include pre- and post-exposure chemoprophylaxis and other HIV treatment services that are currently available or may soon be made available to them.

We observed significantly higher HIV prevalence among MSM in current regular partnerships than among those with non-regular partners. Other data from South Africa show that risk behavior is more likely to take place in partnerships perceived as regular versus non-regular, and that regular partnerships are often not long-lasting. [34], [35] In light of MPMS findings of low prevalence of regular testing and high prevalence of undiagnosed HIV, coupled with previous studies showing that MSM in regular partnerships are not likely to communicate about HIV status, [36] it is unlikely that unprotected sex between partners reflects negotiated safety based on individuals' accurate knowledge of their HIV status and disclosure of that status to a partner. Rather, given the low utilization of regular HIV testing, higher prevalence among those in regular partnerships is more likely indicative of a high probability of HIV transmission within these partnerships. This suggests that interventions focused on communication between sexual partners about condom use and testing for HIV, including testing for HIV as a couple, may potentially be of great benefit to MSM. Increasing efficacy for communication between partners about condom use and HIV testing, including couples testing, are among the prevention behaviors that the adapted Boithato intervention aims to encourage. MSM couples-based testing has been shown to be acceptable to South African MSM, [37], [38] and is available in Mpumalanga, but does not appear currently to be well-utilized by MSM.

The number of MSM in the MPMS baseline who were aware of their HIV-positive status and had linked to care, while well below optimum levels, was comparable to what has been observed generally in the Sub-Saharan Africa region. [39] Among men who were already aware of their HIV infection, the proportion reporting being linked to care was similar to what has been observed among MSM in comparatively higher-income settings in the USA. [40] However, it is unknown whether these participants' post-diagnosis health-seeking behavior is typical for South African MSM. On the basis of other data, we hypothesize that this is not likely to be typical behavior for Mpumalanga MSM; rather, these HIV-positive participants may be relatively empowered individuals who previously sought HIV testing and attended clinics despite social vulnerabilities that are highly prevalent in this population, including low socioeconomic status and limited educational attainment, associated with low uptake of testing, [41] and clinical environments perceived to be unwelcoming to MSM. [18], [19] These MSM have therefore found their way into care despite the lack of social interventions to encourage and support testing and treatment behaviors among MSM, or structural interventions in clinical settings. The baseline assessment provided the initial HIV diagnosis for nearly three-quarters of HIV-infected MSM in this sample. There is a high likelihood that most of these participants will be recaptured in subsequent analysis waves, which will provide a more accurate understanding of post-diagnosis health seeking behaviors, as well as an assessment of whether the social support provided by the Boithato intervention offers additional impetus to successful linkage and engagement in care.

The Boithato intervention aims in part to encourage greater uptake of both regular HIV testing and treatment. In conjunction with the MPMS and Project Boithato, the Anova Health Institute has established a culturally competent standard of care in Mpumalanga for MSM though its Health4Men program (www.health4men.co.za). Follow-up assessments of Project Boithato will consequently measure whether direct exposures to and community diffusion of the Boithato intervention provides additional impetus towards regular utilization of HIV testing, diagnosis of HIV, and linkage and engagement in care in a culturally competent clinical setting.

Limitations of the MPMS include those that pertain to the assumptions of RDS methodology: specifically, that unbiased RDS estimates depend on fulfilling several related assumptions, namely that MSM in each district form a single, networked population; that individuals can accurately assess their personal network size; and that the crude samples were sufficiently representative of the underlying characteristics of the population. The disproportionate number of young men in these crude samples may have biased RDS-adjusted estimates towards characteristics of this subpopulation; in this case, HIV prevalence could be underestimated, and HIV risk behavior and testing behavior overestimated. Moreover, the MPMS offered HIV counseling and testing in a culturally competent setting, and those MSM more motivated to test for HIV may have been overrepresented in the crude sample. [42] In addition, due to structural and operational constraints, the survey measured several key variables normally associated with HIV infection, risk behavior, or health seeking behavior outcomes through self-report that are subject to social desirability bias. It is possible that previous HIV diagnosis, number of partners, and receptive anal intercourse were underreported, and that regular partnerships, consistent condom use, and regular recent HIV testing were over-reported. In addition, our findings on linkage to care are limited by the small numbers of men who self-reported an HIV-positive status and having sought care after their diagnosis prior to MPMS enrolment. The MPMS was not able to independently verify whether any of its HIV-positive participants were previously in care, nor if those diagnosed in the study sought care in the public health service.

The MPMS is, to our knowledge, the first study to report on HIV prevalence, associated risk behaviors, and HIV testing, care and treatment behaviors in an MSM population outside of South Africa's largest urban centers of Cape Town, eThekwini/Durban, Johannesburg, and Tshwane/Pretoria. As such, it may be representative of the state of the HIV epidemic among MSM in more resource-challenged rural provinces, [43] as well as an indication of the state of the national response to the epidemic among MSM outside of these major metropolitan centers. South Africa's current HIV/AIDS National Strategic Plan has set ambitious benchmarks for the HIV response with key affected populations, including MSM, that include halving HIV prevalence, ensuring at least 90% are reached by a social and behavioral change communication strategy to increase demand for testing and treatment, and ensuring at least 80% are engaged in care that includes early ART initiation, all by 2017. [44] The MPMS findings demonstrate a critical need for a comprehensive intervention to bring more MSM into timely, regular, and meaningful contact with HIV prevention, testing, and treatment services. Follow up MPMS waves in 2014 and 2015 will show if Project Boithato's approach to building a healthy, supportive MSM community can add value to government and non-governmental organizations' current efforts to engage MSM in HIV prevention and treatment with culturally competent HIV prevention and care services in Mpumalanga, and potentially elsewhere in South Africa.
